# Supplementary material for: Critical Transitions in Intensive Care Units: A Sepsis Case Study
Source: Sci Rep. 2019 Sep 9;9:12888. doi: 10.1038/s41598-019-49006-2 (PMC6733794; doi:10.1038/s41598-019-49006-2)
Supplement: Supplementary file 1 — Supplementary Information [file 41598_2019_49006_MOESM1_ESM.pdf]

Critical Transitions in Intensive Care Units: A Sepsis Case Study

Pejman F. Ghalati[1,+], Satya S. Samal[1,3,+], Jayesh S. Bhat[1], Robert Deisz[2], Gernot Marx[2], and Andreas Schuppert[1,*]

[1]Joint Research Center for Computational Biomedicine, RWTH Aachen University, 52074 Aachen, Germany
[2]Klinik für Operative Intensivmedizin und Intermediate Care, Universitätsklinikum Aachen, 52074 Aachen, Germany
[3]Currently at: BASF SE, Carl-Bosch-Strasse 38, 67056 Ludwigshafen am Rhein, Germany
[*]schuppert@combine.rwth-aachen.de
[+]these authors contributed equally to this work

SUPPLEMENTARY INFORMATION

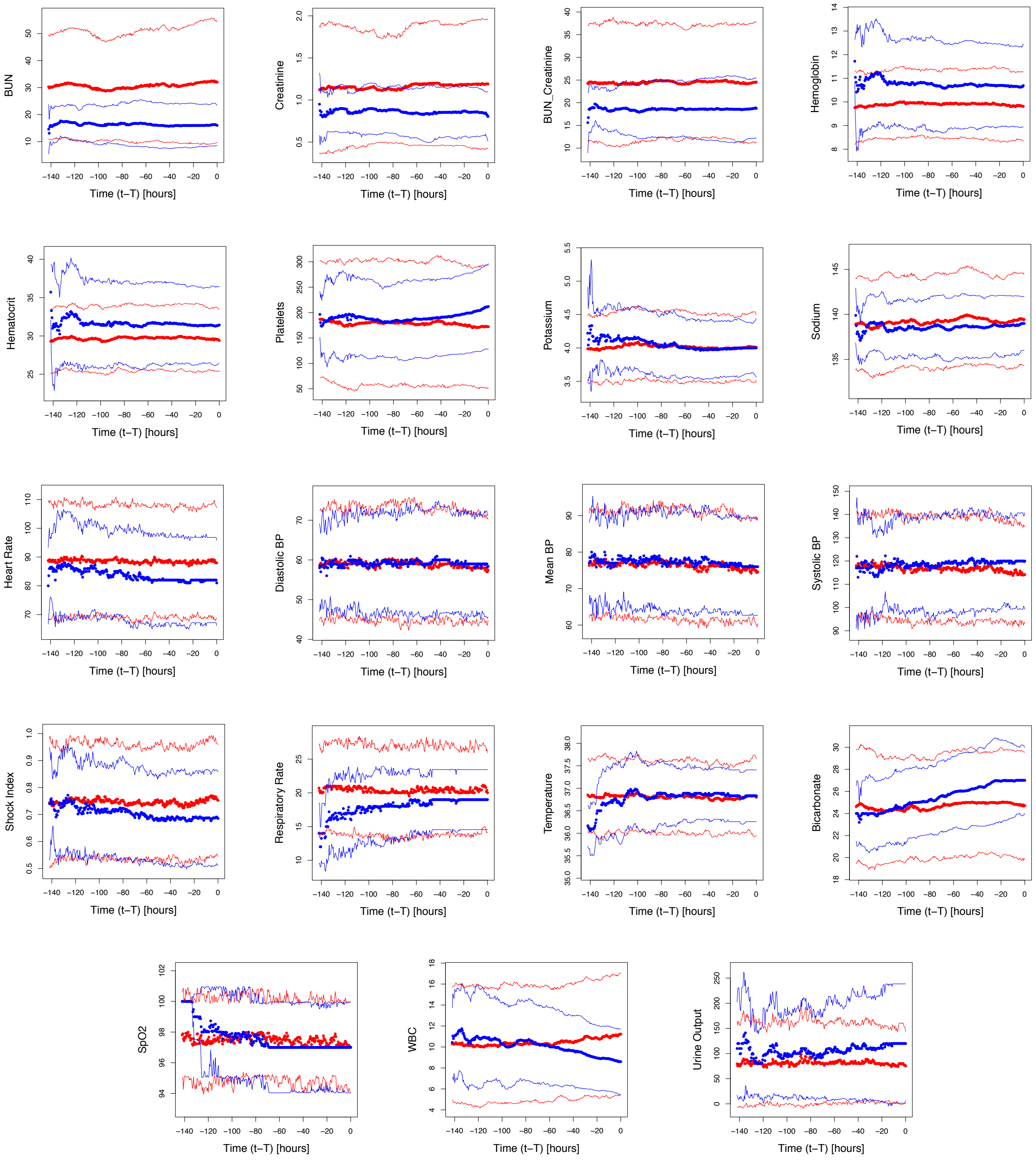

Figure S1. Median (*dots*) and median deviation (*lines*) of variables in septic shock patients (***red***) and non-sepsis patients (***blue***). T is the length of time series, and $t - T$ is the time before the onset of septic shock in septic patients or the time before discharge/decease in non-sepsis patients.

	Non-Sepsis (6236)		Septic Shock (630)	
	Female	Male	Female	Male
Number of patients	2700 (43.29 %)	3536 (56.70 %)	250 (39.68 %)	380 (60.31 %)
Age	63.18 ± 18.76 (185)	60.91 ± 16.03 (86)	65.56 ± 15.18 (4)	64.74 ± 15.38 (4)
In-hospital mortality	629 (23.30 %)	705 (19.94 %)	160 (64.00 %)	248 (65.26 %)
Infectious and parasitic diseases	243 (9.00 %)	305 (8.63 %)	163 (65.20 %)	241 (63.42 %)
Neoplasms	484 (17.93 %)	549 (15.53 %)	49 (19.60 %)	91 (23.95 %)
Endocrine, nutritional and metabolic diseases, and immunity disorders	1783 (66.04 %)	2283 (64.56 %)	189 (75.60 %)	290 (76.32 %)
Diseases of the blood and blood-forming organs	815 (30.19 %)	758 (21.44 %)	151 (60.40 %)	210 (55.26 %)
Mental disorders	864 (32.00 %)	920 (26.02 %)	81 (32.40 %)	98 (25.79 %)
Diseases of the nervous system	659 (24.41 %)	701 (19.82 %)	84 (33.60 %)	124 (32.63 %)
Diseases of the sense organs	223 (8.26 %)	181 (5.12 %)	24 (9.60 %)	23 (6.05 %)
Diseases of the circulatory system	2139 (79.22 %)	2931 (82.89 %)	225 (90.00 %)	345 (90.79 %)
Diseases of the respiratory system	1076 (39.85 %)	1118 (31.62 %)	205 (82.00 %)	313 (82.37 %)
Diseases of the digestive system	885 (32.78 %)	1052 (29.75 %)	164 (65.60 %)	243 (63.95 %)
Diseases of the genitourinary system	705 (26.11 %)	880 (24.89 %)	186 (74.40 %)	278 (73.16 %)
Complications of pregnancy, childbirth, and the puerperium	17 (0.63 %)	0 (0.00 %)	0 (0.00 %)	0 (0.00 %)
Diseases of the skin and subcutaneous tissue	144 (5.33 %)	147 (4.16 %)	50 (20.00 %)	88 (23.16 %)
Diseases of the musculoskeletal system and connective tissue	661 (24.48 %)	405 (11.45 %)	46 (18.40 %)	61 (16.05 %)
Congenital anomalies	128 (4.74 %)	147 (4.16 %)	13 (5.20 %)	13 (3.42 %)
Certain conditions originating in the perinatal period	0 (0.00 %)	0 (0.00 %)	0 (0.00 %)	0 (0.00 %)
Symptoms, signs, and ill-defined conditions	779 (28.85 %)	835 (23.61 %)	145 (58.00 %)	227 (59.74 %)
Injury and poisoning	824 (30.52 %)	1107 (31.31 %)	197 (78.80 %)	289 (76.05 %)
External causes of injury and supplemental classification	1662 (61.56 %)	2114 (59.79 %)	170 (68.00 %)	269 (70.79 %)

Table S1. Demographic data of our septic shock and non-sepsis cohorts. According to MIMIC-III documentation, date of birth has been shifted for patients older than 89. The median age for such *very old* patients was 91.4. When calculating mean age of the individual groups, *very old* patients' age (count of such patients indicated in parenthesis) was set to 91.4. The major categories of disease was based on ICD9

Variable	$-log_{10}(P-value)$		BF	BF (36 hrs)	BF (18 hrs)
	t_{max}	Random Time Points			
BUN	92.2	86.9	0.98	0.05	0.00
BUN-Creatinine	44.2	37.8	1.00	0.99	0.99
Creatinine	36.0	33.3	0.94	0.14	0.00
Hemoglobin	20.2	18.2	0.86	0.81	0.00
Hematocrit	15.1	11.6	0.99	0.99	0.51
Shock Index	14.4	8.9	0.99	0.98	0.77
Respiratory Rate	14.3	12.8	0.75	0.95	0.99
Heart Rate	12.9	11.6	0.75	0.72	0.50
Systolic BP	9.0	2.1	1.00	0.99	0.99
Bicarbonate	6.6	9.4	0.1	0.00	0.00
Platelets	5.3	5.6	0.35	0.00	0.00
Temperature	4.8	1.9	0.99	0.93	0.95
Urine Output	4.6	6.0	0.2	0.00	0.00
WBC	4.0	3.5	0.75	0.00	0.00
Mean BP	2.6	1.1	0.97	0.83	0.52
SpO2	1.9	0.9	0.91	0.00	0.00
Diastolic BP	1.8	1.0	0.85	0.32	0.06
Sodium	1.2	1.3	0.42	0.00	0.00
Potassium	0.6	0.2	0.82	0.92	0.98
SLMean	19.3	-	-		

Table S2. A statistical significance test (see Sect. 2.4) was performed to test whether the values of clinical variables at largest $SLMean$ were able to statistically differentiate septic shock patients from non-sepsis patients. $-log_{10}(P-value)$ of each variable at t_{max} were compared with the median $-log_{10}(P-value)$ of randomly selected points. The last three columns show the corresponding *BF (Full)*, *BF (36 hours)* and *BF (18 hours)* of each variable.

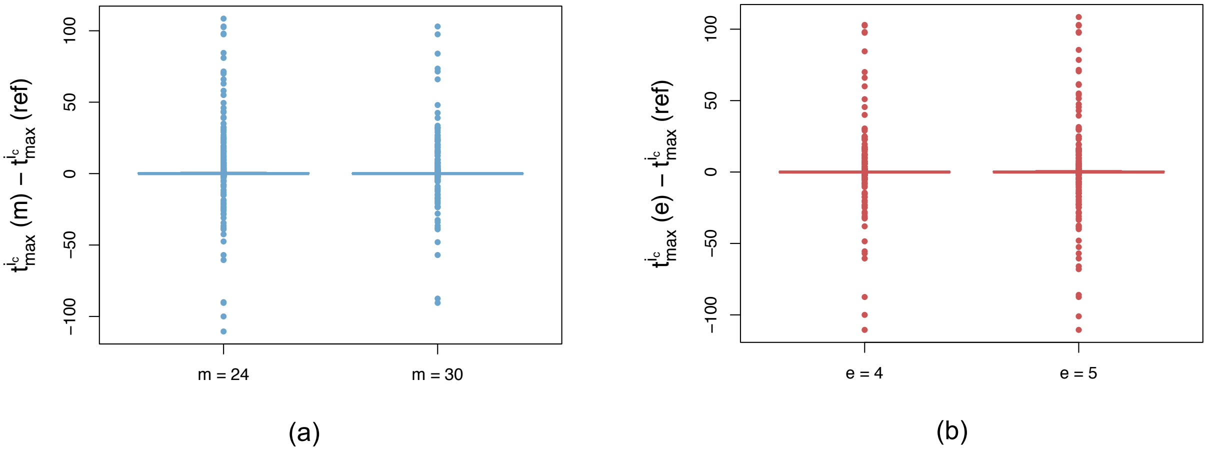

Figure S2. The robustness of our procedure was examined by calculating SL of septic patients ($c = 1$ in Sect. **??**) using different settings of moving time-window ($m \in (24, 30)$ and $e \in (4, 5)$). The corresponding $t^{i_c}_{max}$ for each set of parameters, along with their difference from the reference parameter set ($m = 36$, $e = 3$), were obtained. a) The difference between $t_{max}(m)$ and $t^{i_c}_{max}(ref)$ shows the effect of moving time-window length on $t^{i_c}_{max}$. b) The difference between $t^{i_c}_{max}(e)$ and $t^{i_c}_{max}(ref)$ shows the effect of number of trends on $t^{i_c}_{max}$.
